# Colonic Inflammation in Mice Is Improved by Cigarette Smoke through iNKT Cells Recruitment

**DOI:** 10.1371/journal.pone.0062208

**Published:** 2013-04-25

**Authors:** Muriel Montbarbon, Muriel Pichavant, Audrey Langlois, Edmone Erdual, François Maggiotto, Christel Neut, Thierry Mallevaey, Sébastien Dharancy, Laurent Dubuquoy, François Trottein, Antoine Cortot, Pierre Desreumaux, Philippe Gosset, Benjamin Bertin

**Affiliations:** 1 Université Lille Nord de France, Lille, France; 2 Inserm U995, F-59045 Lille, France; 3 LI3- Team 8, Centre d’Infection et d’Immunité de Lille; Institut Pasteur de Lille, Lille, France; 4 Inserm U1019, Lille, France; 5 CNRS, UMR 8204, Lille, France; 6 Department of Immunology, University of Toronto, Toronto, Canada; 7 CHU Lille, Service des Maladies de l’Appareil Digestif et de la Nutrition, Hôpital Claude Huriez, Lille, France; 8 UDSL, Faculté des Sciences Pharmaceutiques et Biologiques, Lille, France; University of Auvergne, France

## Abstract

Cigarette smoke (CS) protects against intestinal inflammation during ulcerative colitis. Immunoregulatory mechanisms sustaining this effect remain unknown. The aim of this study was to assess the effects of CS on experimental colitis and to characterize the intestinal inflammatory response at the cellular and molecular levels. Using the InExpose® System, a smoking device accurately reproducing human smoking habit, we pre-exposed C57BL/6 mice for 2 weeks to CS, and then we induced colitis by administration of dextran sodium sulfate (DSS). This system allowed us to demonstrate that CS exposure improved colonic inflammation (significant decrease in clinical score, body weight loss and weight/length colonic ratio). This improvement was associated with a significant decrease in colonic proinflammatory Th1/Th17 cytokine expression, as compared to unexposed mice (TNF (p = 0.0169), IFNγ (p<0.0001), and IL-17 (p = 0.0008)). Smoke exposure also induced an increased expression of IL-10 mRNA (p = 0.0035) and a marked recruitment of iNKT (invariant Natural Killer T; CD45+ TCRβ+ CD1d tetramer+) cells in the colon of DSS-untreated mice. Demonstration of the role of iNKT cells in CS-dependent colitis improvement was performed using two different strains of NKT cells deficient mice. Indeed, in Jα18KO and CD1dKO animals, CS exposure failed to induce significant regulation of DSS-induced colitis both at the clinical and molecular levels. Thus, our study demonstrates that iNKT cells are pivotal actors in the CS-dependent protection of the colon. These results highlight the role of intestinal iNKT lymphocytes and their responsiveness to environmental stimuli. Targeting iNKT cells would represent a new therapeutic way for inflammatory bowel diseases.

## Introduction

Ulcerative colitis (UC) and Crohn’s disease (CD) are chronic, relapsing immune-mediated disorders of the gastrointestinal tract of unknown etiology. Emerging evidence suggests that disease development involves a deregulated dialogue between the intestinal flora and components of both the innate and adaptive immune systems in genetically susceptible individuals, under the influence of environmental factors [1], [2]. The genetic contribution is now well characterized as genome wide association studies (GWASs) have identified a number of susceptibility genes that predispose to CD and/or UC [3]. Environmental factors likely affect the incidence and disease history of inflammatory Bowel diseases (IBD) and among them active smoking has been established as the most robust risk factor [4], [5].

However, the effect of smoking appears to be ambivalent: smoking was shown to double the risk of developing CD and to worsen its course, increasing the need for steroids, immunosuppressants, and surgery [4], [5]. On the contrary, smoking has been described as protective against UC (UC is 2.5 less frequent in smokers) and, after disease onset, improves its course, decreasing the frequency of flare-up episodes, the need for steroids, and the colectomy rate [4], [5]. Finally it has been established that smoking cessation improves CD and worsens UC.

In contrast to this well-established relationship between IBD and tobacco, few experimental works have been undertaken in order to explore the role of cigarette smoke (CS) on intestinal homeostasis and to date, the underlying mechanism(s) of the effect of smoking in IBD still remain(s) unclear. It appears to be complex, probably involving different substances, including at least nicotine, oxygen free radicals, and carbon monoxide, and acting on different hypothetical targets such as mucus layer, cytokine and eicosanoid production, immune cell functions, gastrointestinal motility and microvasculature [5]–[7]. Despite some limitations, animal models of chemically-induced colitis are widely used to study intestinal homeostasis and pathophysiology of IBD and some of them have been used to study the effect of CS (or one of its component) on the development of intestinal inflammation giving conflicting results [8]. For example, Galeazzi *et al.* described an aggravation of DNBS-induced colitis by CS exposure and nicotine administration in rat [9], whereas a few years later, Ko *et al.* reported beneficial effects of CS inhalation and nicotine administration in the same experimental model [10], [11]. More importantly, most studies are far from accurately reproducing tobacco intoxication since only one CS component (most often nicotine) was administered orally or subcutaneously.

Natural killer T (NKT) cells are a population of T lymphocytes that express NK cell markers and recognise glycolipid antigens presented by the non-classical MHC molecule CD1d [12]. Due to their capacity to produce large amount of cytokines and chemokines, they are potent immunoregulatory cells in various physiological or pathological situations [13], [14]. Invariant (i) NKT cells are a subgroup characterized by their expression of a restricted TCR repertoire composed of an invariant TCR-α (Vα14-Jα18 in mice and Vα24-Jα18 in humans) and are present in the intestine of both humans and mice. Their contributions to the gut homeostasis remain elusive, since they were shown to display both protective and deleterious roles in IBD patients and in murine models of experimental colitis [15], [16]. Moreover iNKT cells are highly sensitive to environmental stimuli, notably to intestinal microbiota [17], [18] and *ex vivo* to CS extract [19].

Based, on the epidemiological and clinical observations that link cigarette smoking and modulation of intestinal inflammation, we hypothesized that identifying the mechanism involved in the effect of CS on colitis might lead to the characterization of a new anti-inflammatory process involved in colon protection. In order to experimentally reproduce the clinical effect of smoking on colonic inflammation, we used the InExpose® System, a smoking device accurately reproducing human smoking habit. The present study investigated the effect of main stream smoke exposure on experimental colitis induced by DSS in C57BL/6 mice with the aim to characterize the colonic inflammatory response both at the cellular and molecular levels.

## Materials and Methods

### Ethic Statement

Animal experiments were performed in the accredited Institution of Pasteur animal care facility (Institut Pasteur de Lille, France) according to governmental guidelines and approved by the “Comité d’Ethique en Expérimentation Animale Nord-Pas de Calais” (CEEA n°75; ethic committee for animal experimentation of the region Nord-Pas de Calais – France; number of protocol acceptance: CEEA 012012).

### Animals

Specific pathogen-free male C57BL/6 mice (7–8 weeks old) were obtained from Janvier Company (France) and Jα18^−/−^ and CD1d^−/−^ mice (7–11 weeks old) were obtained from the animal facility of the Pasteur Institute in Lille (France). Mice were fed with a standard laboratory diet and given autoclaved tap water *ad libitum*. They were kept in an air-conditioned room with controlled temperature (22±1°C), humidity (65–70%), and day/night cycle (12 h light, 12 h dark). Mice were acclimatized for 1 week before entering the study. Each group contained 6 to 10 animals. Animals were monitored daily for behavior, aspect alteration and body weight loss. Animals presenting signs of suffering (weight loss >20%, prostration, tremors…) were instantaneously euthanized.

### Cigarette Smoke Exposure

Research cigarettes (3R4F reference cigarette, purchased from the University of Kentucky) were used throughout the experiment. A maximum of 20 mice were placed in the ventilated smoking chamber of InExpose® System (Scireq Inc) (see Fig. S1) and exposed to the mainstream smoke of 5 cigarettes (8 puffs/cigarettes, 1 puff of 3 seconds/minutes), five days a week.

### Experimental Colitis

**Figure 1:**
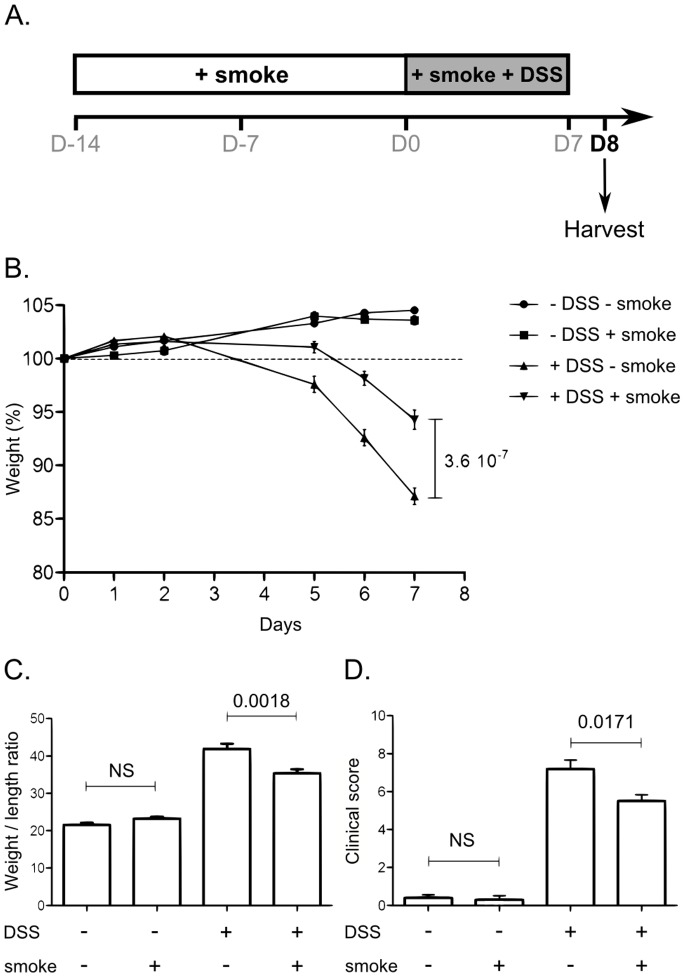
Effect of CS exposure on clinical parameters of colitis induced by DSS. A. Overview of the protocol for CS exposure and experimental colitis. Mice were exposed to CS once a day for three weeks using InExpose® System (Scireq Inc). During the third week, mice were fed 2.5% DSS in their drinking water for 7 days. Mice were killed at day 8. B. Mice body weight changes during induction of colitis. Body weight changes were calculated by dividing body weight on the specified day by the weight of the starting day (day 0) and expressed in percent. 25<n<30; error bars represent SEM. Number on the graph represents p value. C. Colon weight/length ratio represented in mg per cm of colon. Colon were excised from anus to caecum, measured and emptied before being weighed. Graph represents the mean value and error bars represent SEM (17<n<19). Number on the graph represents p value. D. Clinical scores were established according to stool consistency, body weight loss at day 8 and colonic weight/length ratio. Graph represents the mean value of the clinical score and error bars represent SEM (n = 10). NS, non significant. Number on the graph represents p value.

Colitis was induced during the third week of CS exposure and mice were sacrificed at day 8 following colitis induction. Control animals were either untreated or only received DSS or CS alone (see Fig. 1A for detailed protocol).

Colitis was induced by the administration of DSS (molecular weight 40000, TdB consultancy). 2.5% DSS were resuspended in autoclaved tap water and given *ad libitum*. Fresh DSS solution was prepared daily. Control groups received tap water. Mice were weighed every day through DSS exposition. Mice were euthanized by cervical dislocation. The entire colon was removed from the caecum to the anus, then measured, emptied and weighed. A clinical score ranging from 0 to 10 was used to evaluate the severity of the colitis. Score was defined as follows: loss in body weight (0 = no loss; 1 = 5–10%; 2 = 10–15%; 3 = 15–20%; 4 = >20%), stool consistency (0 = Normal pellets; 1 = slightly loose feces, 2 = Loose feces; 3 = Watery diarrhea), colon weight/length ratio (0 = <25 mg/cm; 1 = 26–35 mg/cm; 2 = 36–45 mg/cm; 3 = >45 mg/cm). Mucosal samples from the lower half of the colon were frozen and stored at −80°C for subsequent analysis of inflammatory marker expression.

### RNA Extraction and qPCR Analysis

Total RNA from colon was isolated from tissue using Nucleospin RNA III kit (Macherey Nagel), according to the manufacturer’s instructions. Total RNA from ileum (10 first cm from the caecum to the stomach) was isolated from total ileum homogenate using Trizol reagent (Invitrogen), according to the manufacturer’s instructions, followed by DNAse I (Invitrogen) digestion.

First strand cDNA was synthesized from 1 µg total RNA using High Capacity cDNA Reverse Transcription Kit (Applied Biosystems). Real-time polymerase chain reaction (RT-PCR) was performed using Power SYBR® Green PCR Master Mix (Applied Biosystems) and primers described in table S1. Expression levels of each gene were normalized using β-actin gene expression, yielding the relative expression value.

### Preparation of Intestinal Cells

Intestinal cells from mice were prepared by classical procedures. Colons and liver were excised and finely minced, followed by two enzymatic digestions for 30 min at 37°C in RPMI 1640 containing 1 mg/ml collagenase type VIII (Sigma Aldrich) and 1 µg/ml DNase type I (Sigma Aldrich). After wash, homogenates were resuspended in a 20% Percoll gradient and centrifuged at 2000 rpm, without brake, at room temperature for 10 min. After centrifugation, pelleted cells were aspirated and washed in PBS 2% FCS. RBCs were removed with lysis buffer (Sigma Lysis).

### Flow Cytometry

Cells were prepared as previously described, and stained for 30 min at 4°C with the following Abs: mAbs against mouse CD5 (FITC-conjugated), NK1.1 (PerCp-Cy5.5–conjugated), TCR-β (V450-conjugated), CD45 (Q-dot605-conjugated), and isotypes controls were purchased from Biolegend (Ozyme, Saint-Quentin en Yvelines, France). PE-conjugated PBS57-loaded CD1d tetramer was from the National Institute of Allergy and Infectious Diseases Tetramer Facility (Emory University, Atlanta, GA). mAbs against mouse CD4 (APC-H7-conjugated), CD11c (PE-CY7-conjugated), CD11b (V450-conjugated), Ly6G (Alexa-700-conjugated) and F4/80 (PE-conjugated) were purchased from BD Biosciences (Le Pont de Claix, France). Cells were acquired and analyzed on a Fortessa flow cytometer (Becton Dickinson, Rungis, France), and using the FlowJo software respectively. Gating strategy for the different cell populations is described in Fig. S2.

### Statistical Analysis

Statistical analyses were performed using Prism 4 (GraphPad Software, San Diego, CA) (nonparametric Mann-Whitney test). Differences were considered statistically significant when p value was <0.05. All data were expressed as mean ± SEM or SD.

## Results

### Cigarette Smoke Exposure Improves DSS-induced Colitis

We have first evaluated the impact of CS exposure on the severity of DSS-induced colitis in C57BL/6 WT mice. A standard protocol of exposure was defined and used throughout this study (Fig. 1A). Mice were exposed for three weeks to CS (InExpose® System, Fig. S1). Colitis was induced during the third week (2.5% DSS in drinking water).

As expected, mice treated with DSS alone (DSS-smoke) developed colitis as assessed by body weight loss (Fig. 1B), the increased weight/length ratio of the colon (Fig. 1C) and the measurement of clinical score (Fig. 1D). CS exposure alone had no effect on these parameters. DSS and CS co-treated (DSS+smoke) mice displayed a milder colitis, compared to DSS-smoke mice, as characterized by a delayed body weight loss (Fig. 1B, *p = 3.6×10^−7^*), a reduced colonic weight/length ratio (Fig. 1C, *p = 0.0018*) and clinical score (Fig. 1D, *p = 0.0171*). Histological analysis was performed on samples taken 1 cm above the rectum, but no difference of histological score (as described by Dieleman [20]) was observed between groups (data not shown).

### Cigarette Smoke Exposure Decreases Pro-inflammatory Cytokine Expression during Colitis

**Figure 2:**
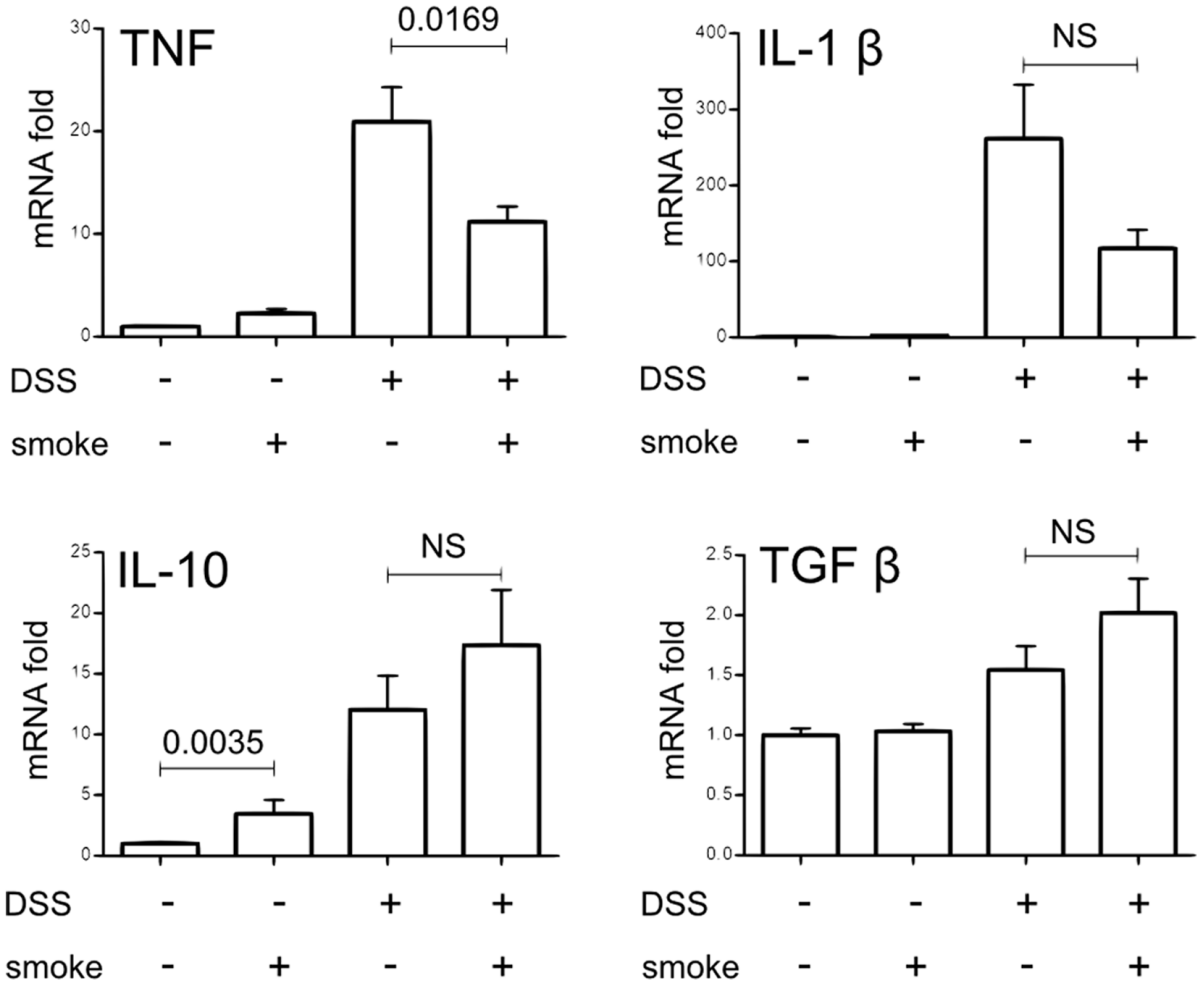
Effect of CS exposure on colonic pro-inflammatory and anti-inflammatory cytokine expression induced by DSS. Cytokine expression in colon homogenates was determined by real time qPCR analysis and normalized by the β-actin expression. Graph represents the mean of the fold expression of each cytokine with the expression level measured to control animals (no CS exposure, no DSS) used as a reference and set to one. Data are pooled from two independent experiments with a total of 17–20 mice/group; error bars represent SEM. NS, Non significant; Number on the graph represents p value.

To further characterize the inflammation, we have analyzed the mRNA expression of cytokines in intestinal tissue homogenates. DSS treatment increased the expression of colonic TNF and IL-1β by over 20 and 200 fold respectively (Fig. 2). Exposition to CS reduced the expression of DSS-induced TNF in the colon (Fig. 2, *p = 0.0169*) whereas the expression of IL-1b tended to be decreased (p = NS). Interestingly, CS exposure alone increased the expression of the IL-10 cytokine (Fig. 2, *p = 0.0035*). Compared to DSS-smoke mice, DSS+smoke mice showed a higher level of IL-10 and TGF-β cytokines in the colon but values were not statistically significant.

**Figure 3:**
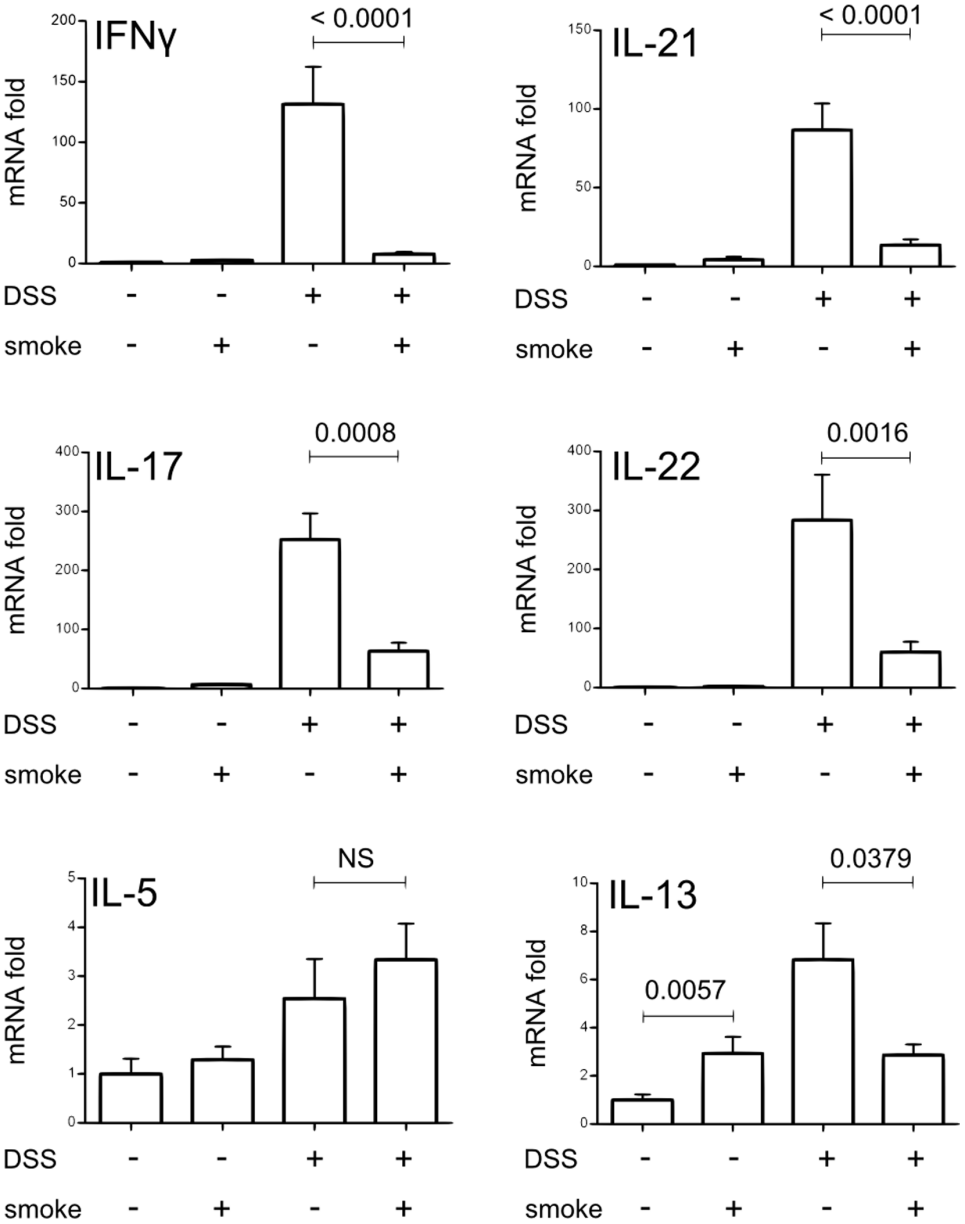
Effect of CS exposure on colonic Th1/Th2/Th17 cytokine expression induced by DSS. Cytokine expression in colon homogenates was determined by real time qPCR analysis and normalized by the β-actin expression. Graph represents the mean of the fold expression of each cytokine with the expression level measured to control animals (no CS exposure, no DSS) used as a reference and set to one. Data are pooled from two independent experiments with a total of 17–20 mice/group; error bars represent SEM. NS, Non significant; Number on the graph represents p value.

DSS treatment strongly increased mRNA expression of Th1/Th17 proinflammatory cytokines IFNγ, IL-21, IL-17 and IL-22 (Fig. 3, fold risen up from 100 to 300), which confirms previous observations [21]. CS exposure drastically decreased the expression of DSS-induced IFNγ (Fig. 3, *p<0.0001*), IL-21 (*p<0.0001*), IL-17 (*p = 0.0008*) and IL-22 (*p = 0.0016*). IL-12/IL-23 sub-unit p40 mRNA expression was also analyzed but no significant difference was observed between the two DSS-treated groups (data not shown). Concerning Th2 cytokines, IL-5 and IL-13 were increased by DSS treatment to a lesser extent (Fig. 3, up to 8-fold increase). IL-13 expression in the colon was up-regulated by CS exposure alone (*p = 0.0057*) and decreased in DSS+smoke compared to DSS-smoke mice (*p = 0.0379*). CS exposure had no effect on IL-5 expression and IL-4 was never detected in the colon of mice (not shown).

Our results demonstrate that CS exposure is able to limit acute colonic inflammation induced by DSS treatment and that this effect is linked to a down-regulation of Th1/Th17 cytokine expression in inflamed colon.

### Cigarette Smoke Protection is Not Linked with Modification in Neutrophil Recruitment or Activation in the Colon

**Figure 4:**
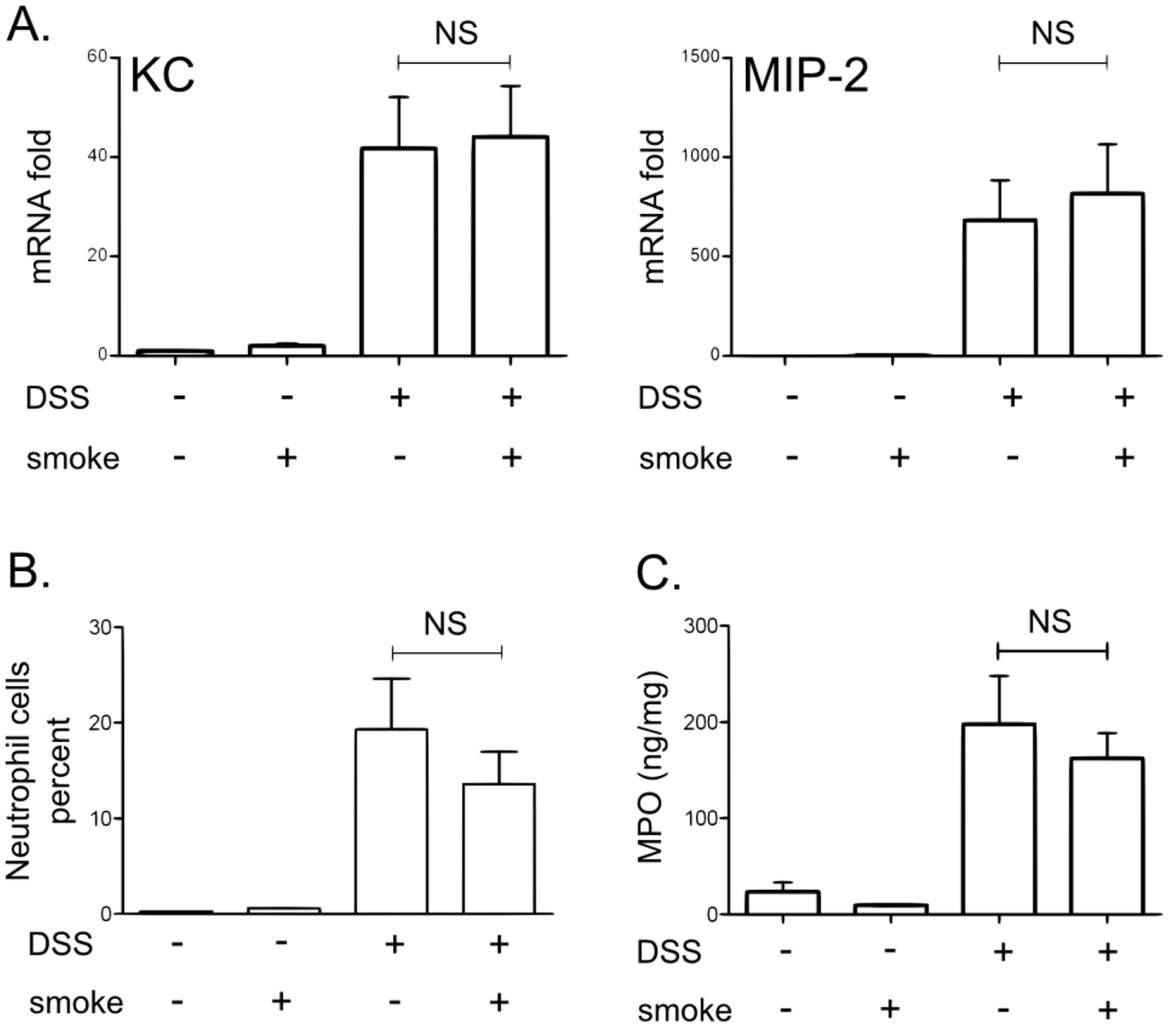
Effect of CS exposure on neutrophils recruitment and activation induced by DSS colitis. A. CXCL1/KC and CXCL2/MIP-2 mRNA expression in colon homogenates was determined by real time qPCR analysis and normalized by the β-actin expression. Data are pooled from two independent experiments with a total of 17–20 mice/group; error bars represent SEM. NS, Non significant; Number on the graph represents p value. B. Mean ± SD of neutrophil (CD11c− Ly6G+) percentages of CD45+ cells in the colonic *lamina propria* (n = 5). NS, non significant**.** C. Colonic MPO level determined by ELISA. The graph represents the mean ± SD of the final values of MPO level expressed in ng/mg of protein (n = 10). NS, non significant.

Neutrophils constitute one of the most prominent infiltrating cells and have been suggested to contribute significantly to the generation of tissue injury in intestinal inflammation [22], [23]. It is therefore logical to assume that neutrophil recruitment could be decreased by CS exposure and could lead to the protecting effect observed in our model. We therefore evaluated CXCL1/KC and CXCL2/MIP-2 mRNA expression, two critical effectors for neutrophil trafficking. Their expression were up-regulated by DSS treatment but were not modulated by CS exposure (Fig. 4A) suggesting that neutrophil recruitment was not impacted by CS. Similarly, DSS-induced colitis was associated with an increase in colonic neutrophil infiltration (Fig. 4B) and myeloperoxidase (MPO) levels (Fig. 4C) which were not modulated by CS.

The decrease of inflammation associated with CS exposure in DSS treated mice is not linked to a modification in neutrophil recruitment or activation.

### Cigarette Smoke Modulates iNKT Cell Numbers in the Colon and the Liver

**Figure 5:**
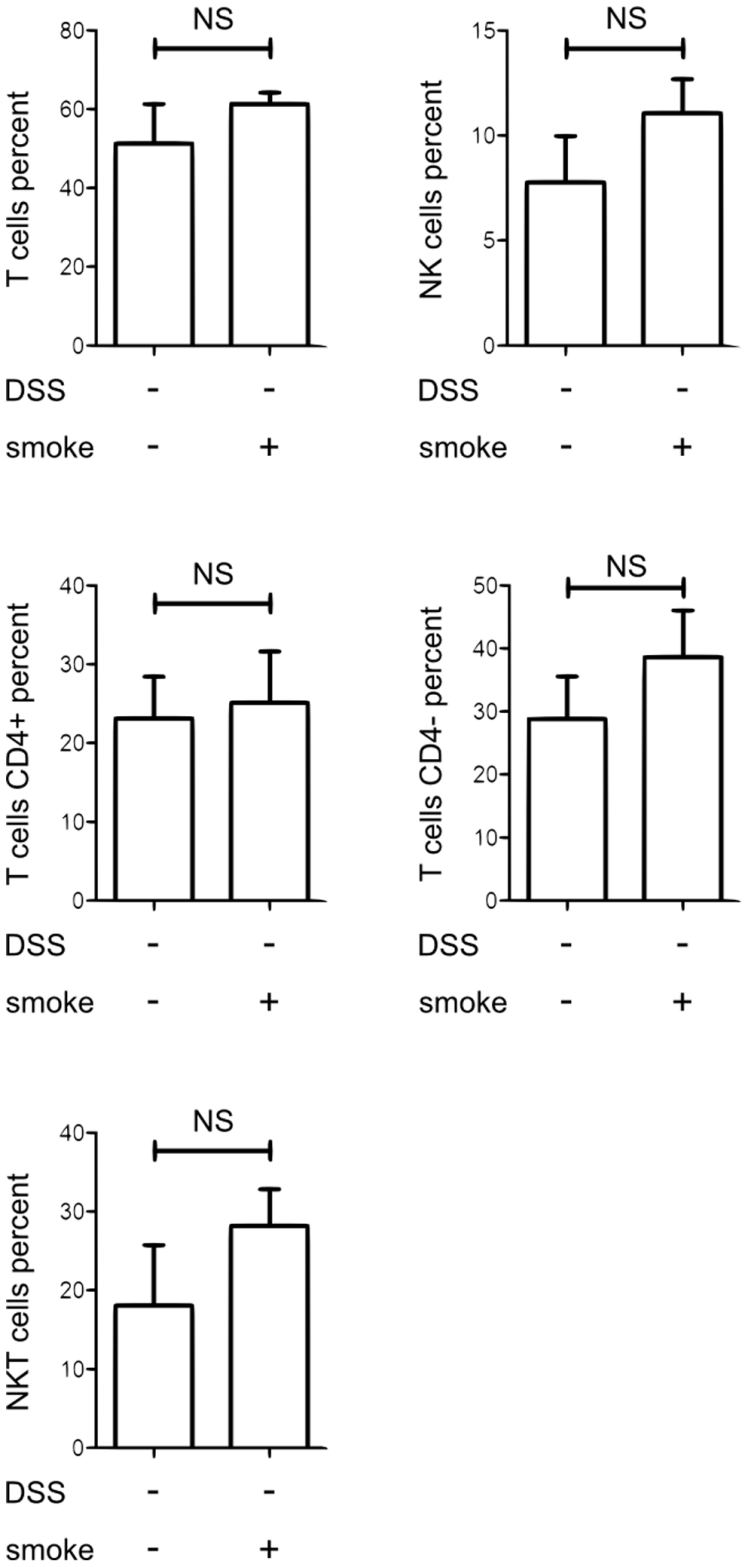
Effect of CS exposure on colonic cell recruitment. The percentages of lymphocyte cell population (conventional T cells, NK cells and total NKT cells) among CD45+ cells were represented in the colonic mucosa of mice exposed or not to CS (20 mice/groups). Colonic tissues of 5 mice of the same exposure group were pooled before cell extraction in order to obtain enough cells for cytometry analysis. Graph represents the mean value ± SD of the cell percentage in the colonic mucosa according to CS exposure. Number on the graph represents p value.

**Figure 6:**
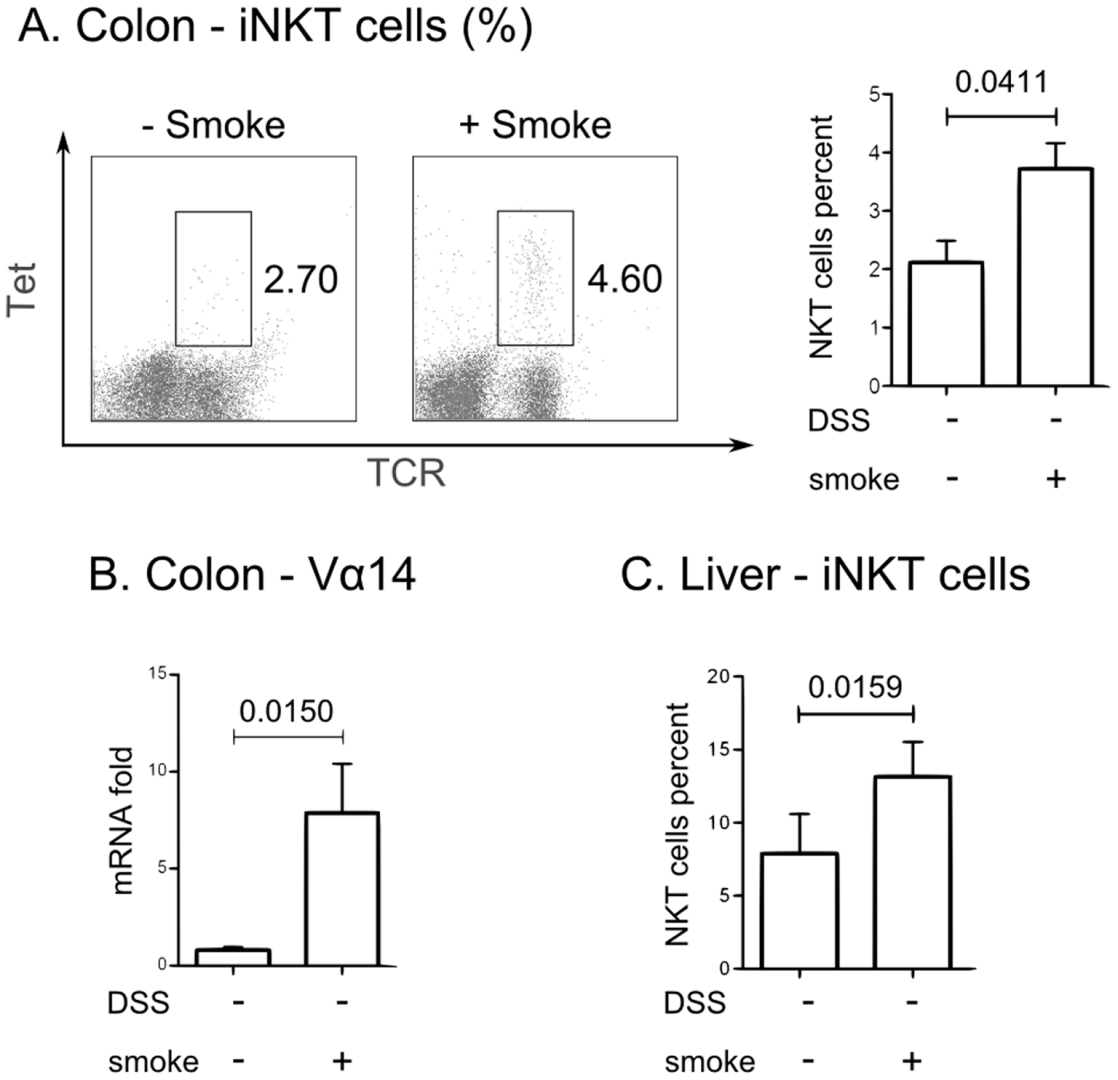
Effect of CS exposure on NKT cell recruitment. A. Percentage of iNKT cells (TCRβ^+^ CD1d tetramer^+^) in CD45^+^ cells in the colonic mucosa of mice exposed or not to CS. Colonic tissues of several mice per group (3 to 5 animals) were pooled before cell extraction in order to obtain enough cell for cytometry analysis. On the left: a representative dot plot is shown. Numbers indicate iNKT cell percentage. On the right: Graph represents the mean value ± SEM of the iNKT cell percentage in the colonic mucosa according to CS exposure (n = 32 mice/condition from 3 independent experiments). Number on the graph represents p value. B. Vα14 mRNA expression in the colon as determined by real time qPCR analysis after normalization by the β-actin expression (17<n<20). The expression level measured with control animals un-exposed to CS was set to one. Graph represents mean value ± SEM. Number on the graph represents p value. C. iNKT cells (TCRβ+ CD1d tetramer+) represented in percent of CD45+ cells in the liver of mice exposed or not to CS for 3 weeks (n = 5). Graph represents the mean value ± SD. Number on the graph represents p value.

In order to identify a particular cell population that could be targeted by CS and involved in the protection from colitis, we have analyzed the leukocyte populations in the colon of mice only exposed to CS as compared with air exposed mice. Flow cytometry analysis revealed that CS exposure did not alter T cell (CD5^+^, NK1.1*^−^*), NK cell (CD5*^−^*, NK1.1^+^) nor total NKT cell (CD5^+^, NK1.1^+^) populations in the colon (Fig. 5, gating Fig. S2). Interestingly, iNKT cells (CD45^+^ TCRβ^+^ PBS57-loaded CD1d tetramer^+^; gating Fig. S2) were recruited in the colon by CS exposure (Fig. 6A; *p = 0.0411*).

Since iNKT cells are recognized as key regulators of intestinal homeostasis [24], we further investigated their implication in the CS-protection against colitis.

We confirmed the recruitment of iNKT cells in the colon of CS exposed mice in three independent experiments by flow cytometry (Fig. 6A) and by qPCR measurement of the Vα14Jα18 TCR gene rearrangement, which is specific to iNKT cells. As expected, Vα14 expression was significantly increased in the colon of mice exposed to CS compared to unexposed mice (Fig. 6B, *p = 0.015*). Since the liver constitutes an important pool of iNKT cells, we analyzed if CS could also mobilize these cells in the liver. As shown in Fig. 6C, the proportion of iNKT cells was increased in the liver of CS exposed mice compared to naive mice (Fig. 6C, *p = 0.0159*).

CS exposure was linked with an increased mobilization of iNKT cells in the colon and the liver whereas the numbers of T, NK and total NKT cells were not modulated.

### CS-dependent Colitis Protection is Lost in NKT Cell Deficient Mice

**Figure 7:**
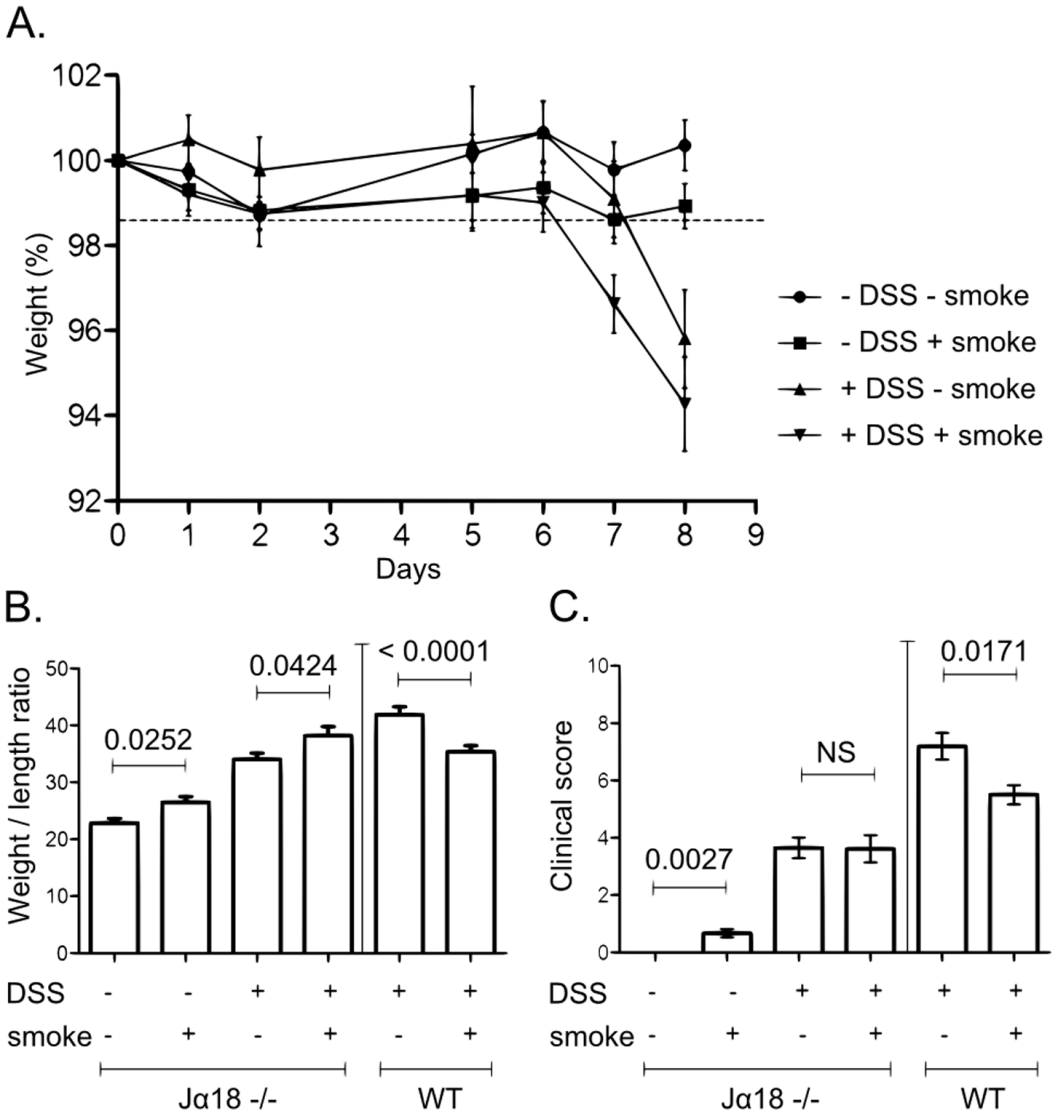
Effect of CS exposure on clinical parameters of DSS-induced colitis in Jα18^−/−^ mice. A. Mice body weight changes during induction of colitis. Body weight changes were calculated by dividing body weight on the specified day by the body weight of the starting day (day 0) and expressed in percent (7<n<12); error bars represent SD. B. Colon weight/length ratio represented in mg per cm of colon. Colon were excised from anus to caecum, measured and emptied before being weighed. Graph represents the mean value and error bars represent SEM (Jα18^−/−^: 9<n<17; WT: 17<n<20). Number on the graph represents p value. C. Clinical scores were established according to stool consistency, body weight loss at day 8 and colonic weight/length ratio (see material and methods). Graph represents the mean value of the clinical score and error bars represent SEM (Jα18^−/−^: 9<n<17; WT: 17<n<20). NS, non significant.

These results led us to test whether iNKT cells were involved in the CS-dependent protection. To address this question, Jα18^−/−^ mice (iNKT cell-deficient mice) were exposed to the same protocol. As shown in Fig. 7, Jα18^−/−^ mice exposed to CS were not protected against DSS colitis. Indeed, DSS+smoke Jα18^−/−^ mice lost weight in an identical manner (Fig. 7A) and displayed the same clinical score (Fig. 7C) than DSS-smoke mice. Moreover, DSS+smoke Jα18^−/−^ mice showed a higher weight/length ratio of the colon than DSS-smoke Jα18^−/−^ mice (Fig. 7B; p = 0.0424).

**Figure 8:**
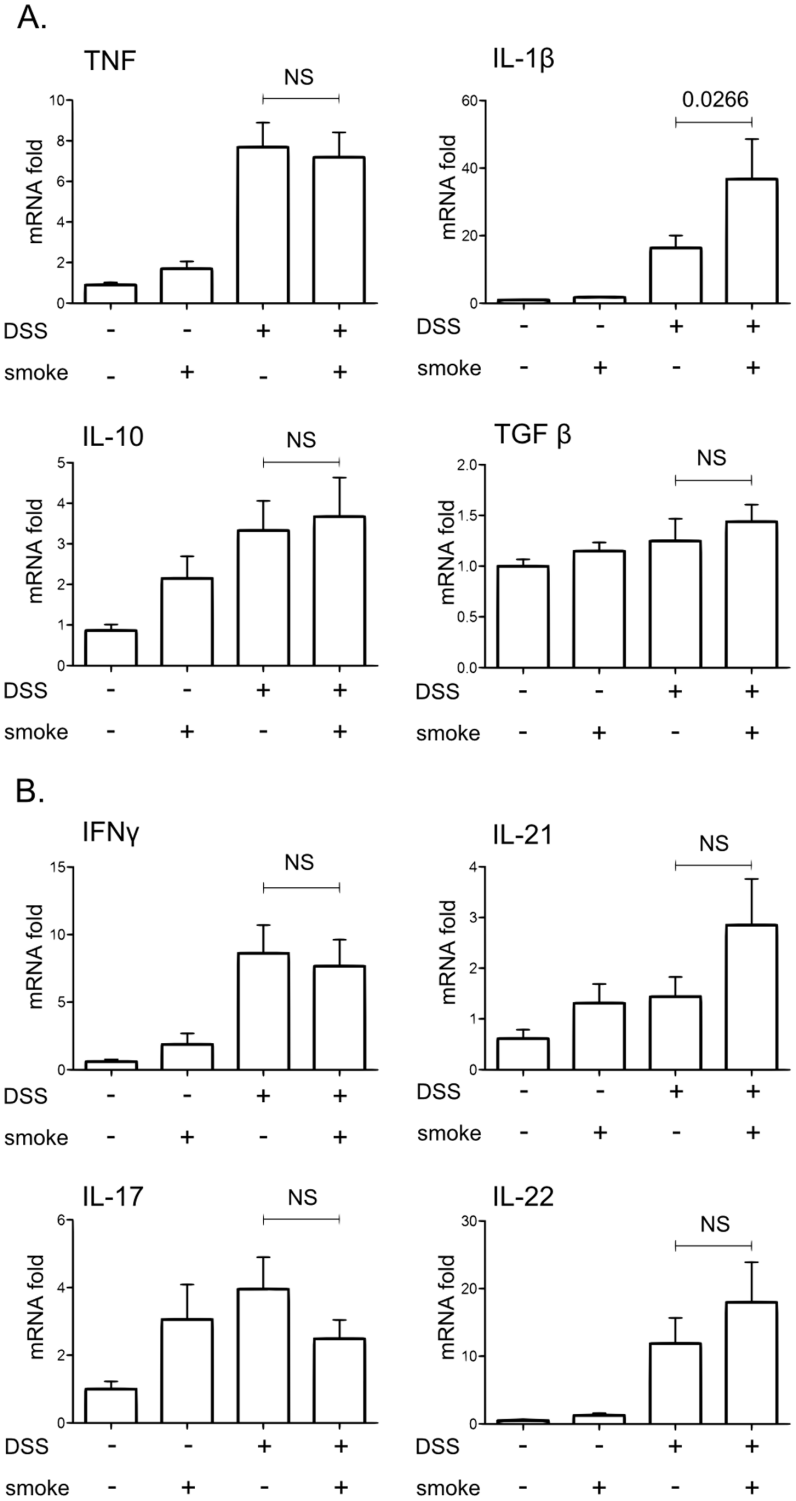
Effect of CS exposure on colonic cytokine expression induced by DSS in Jα18^−/−^ mice. Cytokines expression in colon homogenates was determined by real time qPCR analysis and normalized by the β-actin expression. Graph represents the mean of the fold expression of each cytokine with the expression level measured to control animals (no CS exposure, no DSS) used as a reference and set to one. Data are pooled from two independent experiments (11<n<17). Error bars represent SEM. NS, Non significant; Number on the graph represents p value.

These observations were corroborated by the expression of cytokines in the large intestine. Whereas in WT mice, CS was able to strongly inhibit the expression of colonic proinflammatory cytokines induced by DSS (Fig. 2 and 3), this effect was lost in Jα18^−/−^ mice as revealed by measurement of TNF, IFNγ, IL-21, IL-17 or IL-22 (Fig. 8). IL-1β level was even higher in the colon of DSS+smoke Jα18^−/−^ mice compared to DSS-smoke Jα18^−/−^ mice (Fig. 8A, *p = 0.0266*).

**Figure 9:**
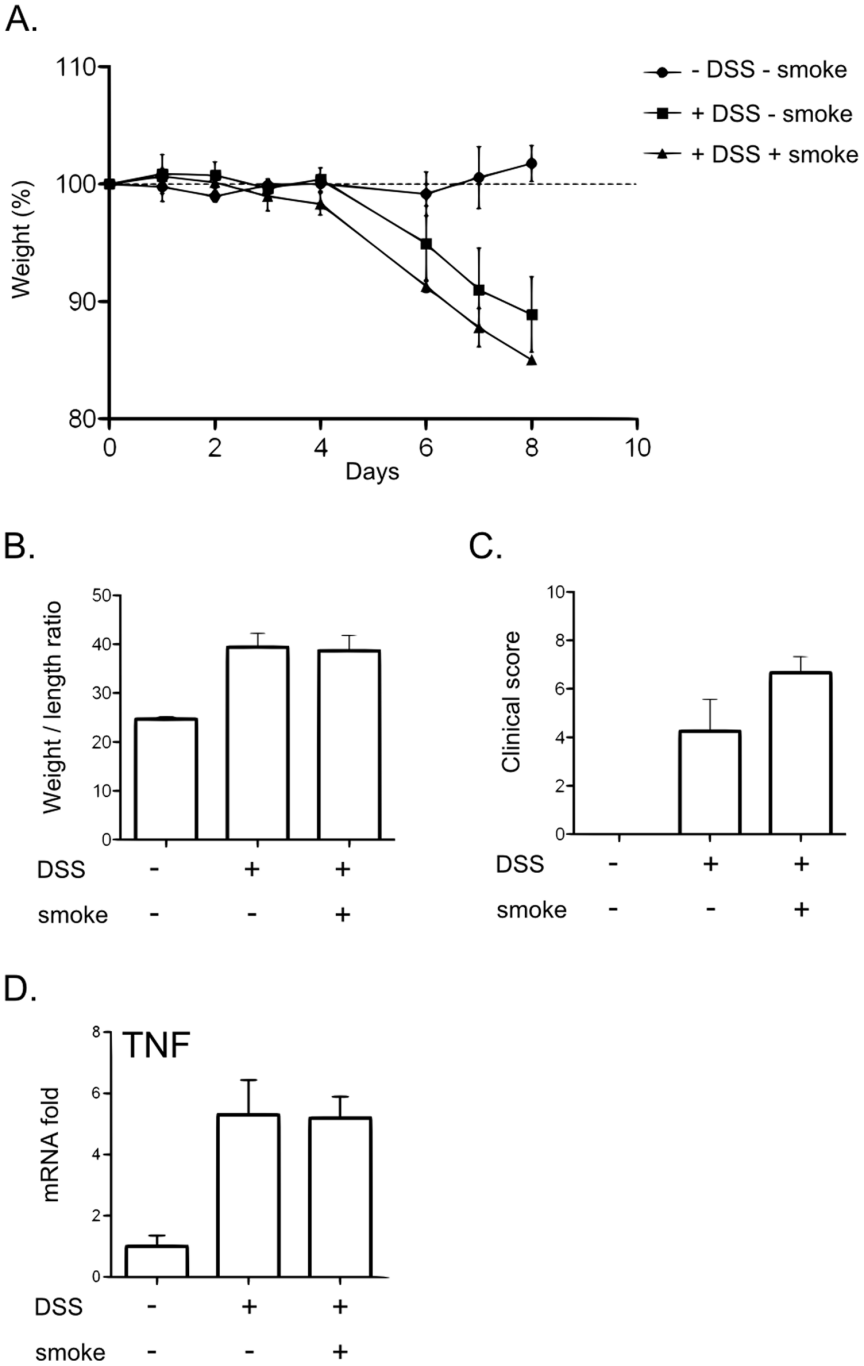
CS exposure did not modulate the development of colitis induced by DSS in CD1d^−/−^ mice. A. Mice body weight changes during induction of colitis. Body weight changes were calculated by dividing body weight on the specified day by the body weight of the starting day (day 0) and expressed in percent (3<n<4); error bars represent SD. B. Colon weight/length ratio represented in mg per cm of colon. Colon were excised from anus to caecum, measured and emptied before being weighed. Graph represents the mean value and error bars represent SD (3<n<4). C. Clinical scores were established according to appearance of diarrhea, body weight loss at day 8 and colonic weight/length ratio (see material and methods). Graph represents the mean value of the clinical score and error bars represent SD (3<n<4). D. TNF expression in colon homogenates was determined by real time qPCR analysis and normalized by the β-actin expression. Graph represents the mean ± SD of the fold expression of TNF with the expression level measured to control animals (no CS exposure, no DSS) used as a reference and set to one (3<n<4).

As activation of iNKT cells is mainly dependent on the presentation of glycolipids by the CD1d molecule, we used CD1d^−/−^ mice to confirm the role of iNKT cells in the CS protective effect. In DSS-treated CD1d^−/−^ mice, CS exposure did not affect clinical parameters (Fig. 9A–C) and TNF expression level analysis in colon homogenates (Fig. 9D).

Altogether, these results show that in DSS-induced colitis, the CS-dependent protection on colonic inflammation is lost in the absence of iNKT cells.

## Discussion

Smoking can influence the risk of emergence and development of IBD with divergent regulatory roles on CD and UC. In this study, we addressed the correlation between smoking and intestinal inflammatory response by evaluating colonic inflammation in mice that have been pre-exposed to CS. We have shown that CS exposure protects the colon from DSS-induced inflammation. Moreover, in an attempt to bring out a particular leukocyte population involved in this process, we have identified the iNKT lymphocytes as a major actor of the CS-dependent protection of the inflammation in the colon. iNKT cells are emerging as an important immunoregulatory population of lymphocytes able to polarize the immune response. iNKT cells are sensitive to environmental stimuli [17], [18] and we showed here for the first time that exposure to an external factor can influence their response in the intestine, bringing a protection against intestinal inflammation.

The effect of cigarette consumption has been investigated in several experimental models of intestinal inflammation, giving conflicting results [8]. Oral or subcutaneous administration of only one CS component is far to reproduce accurately tobacco intoxication. It is also important to distinguish the mainstream smoke (emerging from the filter of a cigarette) and the side stream smoke (emerging from the lit end of the cigarette, *i.e.* passive smoking) which differ significantly [25]. Most studies evaluated the effect of passive CS on intestinal inflammation [10], [11]. To date, only Galeazzi *et al.* examined the role of mainstream smoke on experimental DNBS-induced colitis in rats and described an aggravation of the colitis after CS exposure and oral nicotine administration in rat [9]. These results are not concordant with the human studies reporting a protective effect of CS on the occurrence and course of UC. Therefore, to develop a relevant model, we used the InExpose® System device, which closely mimics human smoking habits. In this chamber, mice were exposed to the mainstream of CS in a rhythmic fashion with the precise control of any desired puffing profile. A period of 2 weeks allowed us to obtain the impregnation of mice and their habituation to smoking. To induce colonic inflammation, we used the recognized model of colitis induced by DSS which mimics some of the molecular, biological and clinical features of UC [21], [26], [27]. This protocol led us to attain a reproducible protective effect of CS exposure on DSS-induced colitis, and to clarify underlying mechanisms.

In this regard, we identified iNKT cells as a central component in CS-induced protection against colitis, highlighting a further role of this cell population within the intestine. In mice, previous studies have shown that NKT cells may exert a protective effect against experimental colitis. Administration of CD1d ligands (α-GalCer and OCH) resulted in a reduction of intestinal inflammation in the colon [28], [29]. Adoptive transfer of NKT cells in TNBS- and in DSS-treated mice alleviated colitis [28], [30]. Finally, two recent studies have provided evidence that iNKT cells are highly sensitive to environmental stimuli, particularly to intestinal microbiota, in order to achieve their maturation [17], [18]. Altogether, these studies are in favor of the control of intestinal tolerance and homeostasis by iNKT cells depending on their location and the inflammatory or environmental context. By demonstrating the iNKT cell involvement in the protective effect of smoking in our model, we now add a new environmental factor that modulates iNKT cell functions in the gut.

By using CD1d tetramer loaded with PBS57 (an αGalcer indistinguishable analog), we found that 2.1±0.36% of *lamina propria* lymphocytes in the colon of control mice were iNKT cells. This result is slightly different from those recently reported by Wingender *et al.* who found a relative percentage of 0.71% using comparable experimental procedure [18]. To our knowledge, no other study has evaluated iNKT cell rates in the colon with CD1d tetramer staining. Therefore, it seems currently difficult to know whether this discrepancy is relevant. One possible explanation could be the origin and the different housing condition of our mice provider (Janvier), since results from the Wingender study clearly showed that variation in the environment of mice between different mice vendors impact iNKT cell number and function [18]. Nevertheless, these data confirm that iNKT cells constitute a significant lymphocyte population within the colon.

Several mechanisms could explain the activation of CS-exposed iNKT cells. The main activation process of iNKT cells involves CD1d-dependent presentation of glycolipids antigens by Antigen Presenting Cells (APC), including intestinal epithelial cells (IEC) [31]. In our model, CS exposure could trigger antigen presentation by IEC resulting in activation and polarization of iNKT cells. Thus, we co-cultured mouse iNKT cells with MODE-K cells (mouse IEC) exposed to soluble CS extract but did not observe any cytokine production by iNKT cells (unpublished data). Another source of CD1d-dependent antigens may be intestinal microflora which may be altered by CS exposure. Even if the role of microbiota on iNKT cells functions is now well established [17], [18], bacterial antigens involved in this process remain to be determined. Finally, iNKT cells have been recently shown to be oriented toward an anti-inflammatory profile by neurotransmitter like noradrenaline [32]. One of the main active components of CS is nicotine, which is a known neurotransmitter protective on UC course. In our model, it could act directly on iNKT cells and lead to an anti-inflammatory profile.

On the other hand, AhR (Aryl hydrocarbon receptor) receptor could play a role in the response of iNKT cells to CS in our model. AhR receptor is a transcription factor implicated in the regulation of inflammatory responses. It binds several pollutants like dioxins [33]. AhR−/− mice are more sensitive to DSS colitis than WT mice while AhR−/+ mice are less sensitive and exhibit a decreased expression of TNF and IL-17 and an increased expression of IL-10 [34] as we observed in our model. Moreover, several recent studies have shown that AhR activation leads to an improvement of DSS colitis [35]–[39], TNBS colitis and oxazolone colitis [37], [40]. As in our own model (Fig. 3), it is remarkable that control of intestinal inflammation through AhR pathway involved a reduction of Th1/Th17 cytokines [33], [37]. This observation appears to be relevant regarding the cytokines profile (Th1/Th17; Fig. 3) regulated by CS in our model. Therefore, activation of AhR by CS components could explain our results. This hypothesis is reinforced by the fact that CS contains significant amounts of dioxin like molecules [41], [42] and is able to activate AhR pathways *in vivo* and *in vitro* [43]. Furthermore, activation of AhR is linked to modifications of number and activation state of NKT cells in the liver [44]. This receptor could therefore play a role in iNKT cell polarization, either directly or indirectly.

Another underlying question is the mechanistic link between iNKT cells and the control of DSS-induced colitis. We showed that mice exposed to CS expressed higher level of IL-10 in their colon than unexposed mice (Fig. 2). To check whether IL-10 was produced by iNKT cells themselves, we stained IL-10 in colonic iNKT cells from smoking and control mice. Unfortunately, we could not detect any change in the intracellular levels of IL-10 when comparing smoking mice with control animals (unpublished data). Another mechanism could be the IL-10 production by other cells orchestrated by iNKT cells after CS stimulation. Indeed, it was recently demonstrated that soluble factors from human iNKT cells have the properties to instruct peripheral blood monocytes to differentiate into suppressive dendritic cells (DC)-like producing IL-10 [45]. Moreover, iNKT cells modulate the immunosuppressive function of IL-10-secreting neutrophils [46]. The capacity of CS-exposed iNKT cells to promote the IL-10 production in both DC and neutrophils should be evaluated in the future.

In conclusion, this study demonstrated that mainstream CS exposure protects mice from experimental colitis and, for the first time, we have identified iNKT cells as a major player of the CS-dependent protection in colonic inflammation. Therefore, our study contributes to better elucidate the impact of smoking, as a widespread environmental factor in IBD. Targeting iNKT cells would represent a novel therapeutic way [47]. Design of new molecules acting on iNKT cell polarization could reproduce the effects of CS and allow to decrease the inflammation in the colon.

## Supporting Information

Figure S1 **Cigarette smoke exposure device InExposure® exposure system (Scireq Inc).** A. General overview. B. Smoking device. C. Exposition chamber.
(TIF)

Figure S2 **Gating strategy for the identification of colonic cells.** A. Gating strategy for iNKT cells (CD45+ CD1d tetramer+ TCR+), total NKT cell (CD5+, NK1.1+), NK cells (CD45+ NK1.1+ CD5−) and conventional T cells (CD45+ NK1.1− CD5+). B. Gating strategy for neutrophils (CD45+ CD11c− CD11b+ Ly6G+ F4/80−).
(TIF)

Table S1 Primers used for PCR analysis. Oligonucleotides sequences of the primers used for Real-time polymerase chain reaction (RT-PCR) analysis.
(DOC)
