# Supplementary figures and images for: Colonic Inflammation in Mice Is Improved by Cigarette Smoke through iNKT Cells Recruitment
Source: PLoS One. 2013 Apr 25;8(4):e62208. doi: 10.1371/journal.pone.0062208 (PMC3636205; doi:10.1371/journal.pone.0062208)

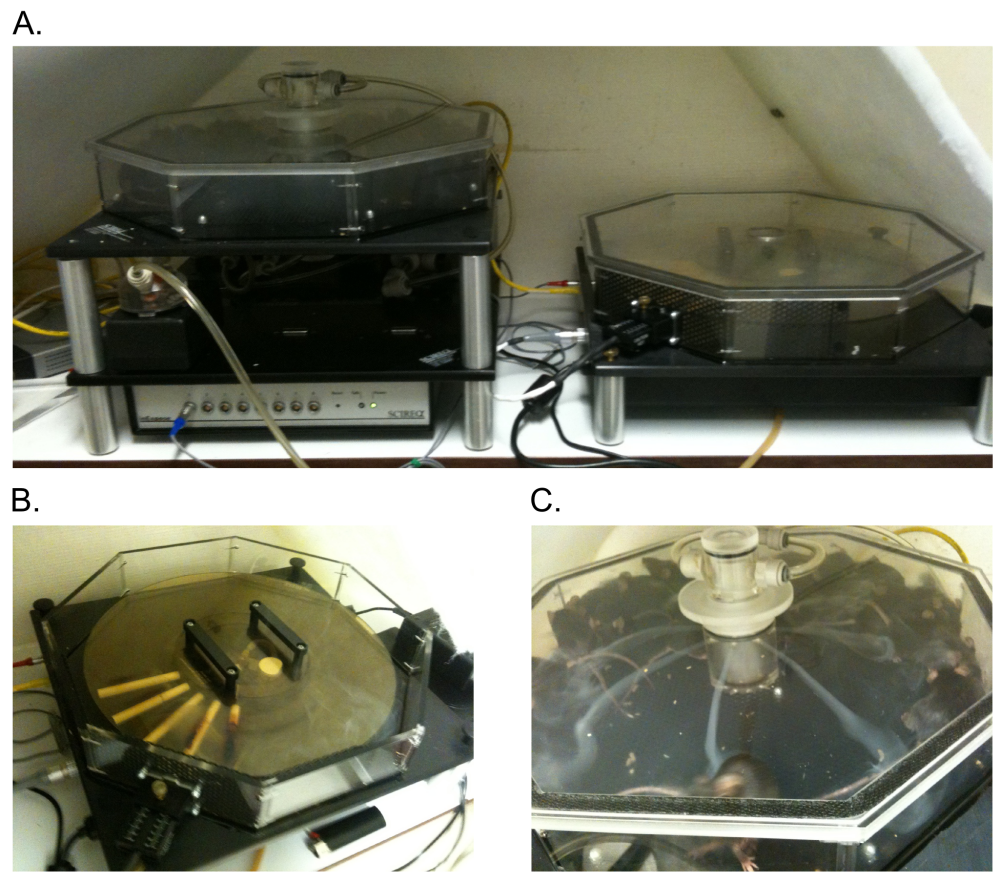

Supplement: Figure S1 — Cigarette smoke exposure device InExposure® exposure system (Scireq Inc). A. General overview. B. Smoking device. C. Exposition chamber. (TIF) [file pone.0062208.s001.tif]
