# Supplementary material for: Colonic Inflammation in Mice Is Improved by Cigarette Smoke through iNKT Cells Recruitment
Source: PLoS One. 2013 Apr 25;8(4):e62208. doi: 10.1371/journal.pone.0062208 (PMC3636205; doi:10.1371/journal.pone.0062208)
Supplement: Table S1 — Primers used for PCR analysis. Oligonucleotides sequences of the primers used for Real-time polymerase chain reaction (RT-PCR) analysis. (DOC) [file pone.0062208.s003.doc]

| **Name** | **Sequence** |
| --- | --- |
| Actin F | TGGAATCCTGTGGCATCCATGAAAC |
| Actin R | TAAAACGCAGCTCAGTAACAGTCCG |
| IFNγ F | ACTGGCAAAAGGATGGTGAC |
| IFNγ R | ACCTGTGGGTTGTTGACCTC |
| IL-10 F | CCCTTTGCTATGGTGTCCTT |
| IL-10 R | TGGTTTCTCTTCCCAAGACC |
| IL-12/IL-23p40 F | GGAAGCACGGCAGCAGAAT |
| IL-12/IL-23p40 R | GGCGGGTCTGGTTTGATG |
| IL-13 F | CAGTCCTGGCTCTTGCTTG |
| IL-13 R | CCAGGTCCACACTCCATACC |
| IL-17A F | GCAAGAGATCCTGGTCCTGA |
| IL-17A R | AGCATCTTCTCGACCCTGAA |
| IL-1β F | CAACCAACAAGTGATATTCTCCATG |
| IL-1β R | GATCCACACTCTCCAGCTGCA |
| IL-21 F | CCTCCTGATTAGACTTCGTCAC |
| IL-21 R | GGTTTGATGGCTTGAGTTTGGC |
| IL-22 F | CAACTTCCAGCAGCCATACA |
| IL-22 R | GTTGAGCACCTGCTTCATCA |
| IL-5 F | GAAGTGTGGCGAGGAGAGAC |
| IL-5 R | GCACAGTTTTGTGGGGTTTT |
| KC F | GGCGCCTATCGCCAATG |
| KC R | CTGGATGTTCTTGAGGTGAATCC |
| MIP-2 F | AGTGAACTGGCGTGTCAATGC |
| MIP-2 R | CCGCCCTTGAGAGTGGCTAT |
| TGFβ1 F | ACCATGCCAACTTCTGTCTG |
| TGFβ1 R | CGGGTTGTGTTGGTTGTAGA |
| TNF F | TGGGAGTAGACAAGGTACAACCC |
| TNF R | CATCTTCTCAAAATTCGAGTGACAA |
| Vα14 F | TGGCTGGCAAGACCCAAG |
| Vα14 R | GCCTGTGTCCTGTTTGAACCAC |
